# Mindful Parenting for Caregivers of Children with Neurodevelopmental Disorder Diagnosis

**DOI:** 10.3390/children13030325

**Published:** 2026-02-26

**Authors:** Pietro Muratori, Alessia Catarina, Anna Conversano, Valentina Levantini, Silvia Villani, Annarita Milone

**Affiliations:** 1 IRCCS Fondazione Stella Maris, 56128 Pisa, Italy; 2 Tabit-Cooperativa Sociale, 53018 Sovicille, Italy; 3 Istituto Beck, 00185 Roma, Italy

**Keywords:** mindfulness, mindful parenting, stress, negative parenting, neurodevelopmental disorders

## Abstract

**Background/Objectives**: The aim of this pilot study was to examine the effect of an eight-session mindful parenting intervention on parents’ mindfulness and parenting dimensions. **Methods**: The intervention was delivered to parents of children with Neurodevelopmental Disorder Diagnosis, followed in a child psychiatry hospital. The sample included 63 adults (40 females; mean age = 44 years). At the beginning and end of the intervention, participants completed questionnaires assessing positive and negative parenting and five mindfulness dimensions. Paired-sample *t*-tests were conducted to detect significant changes in the relevant measures from pre- to post-intervention. **Results**: Results revealed improvements in Negative Parenting (*t* = 2.82, *p* = 0.008, Hedges *g* = 0.29), Acting with Awareness (*t* = −2.14, *p* = 0.039, Hedges *g* = 0.26), Non-Judging (*t* = −2.17, *p* = 0.037, Hedges *g* = 0.35), and Non-Reacting (*t* = −2.68, *p* = 0.011, Hedges *g* = 0.52). **Conclusions**: Despite its preliminary results and single-group pre–post design, this pilot study aligns with evidence supporting mindful parenting interventions and provides initial insights into their impact on parents of children with neurodevelopmental disorders.

## 1. Introduction

Parents play a crucial role in the development and well-being of their children. Parenting represents one of the most demanding and complex roles across the lifespan, requiring continuous emotional, cognitive, and behavioral adaptation to meet children’s evolving needs. Parents are constantly exposed to external demands, including time pressure, emotional labor, and responsibility for their children’s physical and psychological well-being. Parents may experience chronic stress and emotional exhaustion, with significant consequences for their mental and physical health [1]. Prolonged exposure to parenting stress has been associated with increased irritability, reduced emotional availability, and a higher likelihood of engaging in harsh, inconsistent, or coercive parenting practices [2].

These challenges are particularly pronounced for parents of children with neurodevelopmental disorders (ND), such as Attention Deficit Hyperactivity Disorder (ADHD) and Autism Spectrum Disorder (ASD). Children with ND often present persistent difficulties in attention regulation, impulse control, emotional regulation, social communication, and behavioral flexibility, e.g., [3,4,5]. Managing these difficulties in daily life may place a substantial burden on parents who are required to provide high levels of structure, supervision, and emotional support over extended periods of time. Empirical evidence consistently shows that parents of children with ND report significantly higher levels of parenting stress compared to parents of typically developing children, as well as parents of children with other types of chronic conditions [6]. High parenting stress in this population has been linked to maladaptive parenting practices, including increased use of punitive discipline, reduced parental consistency, and difficulties in maintaining positive parent–child interactions. In turn, these parenting behaviors may exacerbate children’s behavioral and emotional difficulties, contributing to a coercive cycle that negatively affects both child outcomes and parental well-being. Interventions aimed at supporting parents’ emotional regulation, stress management, and relational skills are considered a crucial component of comprehensive treatment approaches for children with ND [7,8]. For this reason, we developed the project “*Mindfulness for Parents*” in our child psychiatric hospital, and here we report its preliminary efficacy data.

In recent decades, mindfulness-based interventions have gained increasing attention as effective tools for promoting psychological well-being and self-regulation. Mindfulness is *“the awareness that emerges through paying attention, on purpose, in the present moment, and non-judgmentally to the unfolding of experience moment by moment”* [9] By fostering an open, accepting, and non-reactive attitude toward internal experiences, mindfulness practices are thought to enhance emotional awareness, attentional control, and adaptive coping.

Within the parenting domain, the application of mindfulness principles has led to the development of the mindful parenting construct [10,11]. Mindful parenting encourages parents to bring intentional, non-judgmental awareness to parent–child interactions, fostering a more attuned and compassionate relational stance. Mindful parenting encourages parents to observe their children with attention and interest, without judgment, focusing on the “*here and now*” and reducing automatic and reactive responses. According to the model proposed by Duncan et al. [10], mindful parenting encompasses five core dimensions: listening with full attention, emotional awareness of self and child, self-regulation in the parenting relationship, non-judgmental acceptance of self and child, and compassion for self and child. Through these processes, mindful parenting aims to reduce automatic and reactive responses and promote more flexible, intentional, and supportive parenting behaviors.

A substantial body of empirical evidence has examined the efficacy of Mindfulness-Based Interventions (MBIs), such as MBSR and MBCT, for parents of children with ND, including ASD and ADHD. These studies consistently showed that mindfulness practice can significantly reduce parental stress and improve parental awareness and well-being, and children’s symptoms, e.g., [12,13,14]. Despite the increasing empirical research, less is known about the effects of mindful parenting interventions. While MBIs primarily focus on parents’ individual psychological states and stress reduction, with parenting representing a secondary outcome, mindful parenting interventions explicitly aim to modify parents’ moment-to-moment awareness, emotional reactivity, and relational responses within parent–child interactions. Given the unique challenges faced by families with children with ND, mindful parenting programs might represent a promising option as they might more narrowly address the emotional and relational demands associated with these conditions and better support parents in enhancing self-regulation and responding to their children’s needs with greater awareness and compassion.

Overall, systematic reviews and meta-analyses suggest that mindful parenting is associated with lower levels of parenting stress and psychological distress, as well as more adaptive parenting practices across different populations [15,16]. Moreover, mindful parenting has been linked to fewer internalizing and externalizing symptoms in children, improved emotion regulation, and better social functioning [17] as well as small but significant improvements in parental mindfulness, parenting stress, and child behavior [18]. However, the majority of studies included in these reviews have been conducted in non-clinical samples or different clinical populations (e.g., children with medical conditions or more general behavioral problems), with relatively few focusing on parents of children with ND [19,20].

Based on these considerations, the present pilot study aimed to evaluate the efficacy of a mindful parenting intervention specifically designed for parents of children with neurodevelopmental disorders, including ADHD and ASD, with or without comorbid Specific Learning Disorder (SLD). Taking into account evidence highlighting that intervention characteristics, such as program duration and tailoring to specific populations, may influence intervention efficacy, with longer and more targeted programs yielding stronger effects [21,22], the program was specifically adapted to help parents of children with neurodevelopmental disorders increase awareness, emotional regulation, and compassionate responses in challenging parenting situations. It consisted of eight weekly two-hour sessions and included formal mindfulness practices, experiential exercises, psychoeducation and group discussions. The primary objectives were to preliminarily examine changes in parenting practices (i.e., positive and negative parenting) and in the five facets of dispositional mindfulness from pre- to post-intervention.

## 2. Materials and Methods

### 2.1. Participants and Procedure

Parents of children followed at the “Fondazione Stella Maris”—a specialized scientific hospital that delivers diagnostic and clinical child psychiatry services to families referred from all regions of Italy—were recruited through an online announcement published on the Institute’s website. Parents were eligible if their child was between 5 and 12 years old and had a diagnosis of a neurodevelopmental disorder (i.e., ADHD and ASD, with or without comorbid SLD). They could participate either as a couple or individually. Between January 2024 and June 2025, a total of 23 fathers and 40 mothers (mean age = 44 years and 2 months) initially joined the program. After the first two meetings, 13 (20%) parents dropped out, leaving 50 participants who comprised the analytic sample. This attrition rate is similar to the rate reported in previous reviews [17]. Ultimately, the groups comprised the parents of 36 children (mean age = 8 years and 7 months). Among them, 18 had a primary diagnosis of ADHD, 18 of ASD, and 22 also had comorbid SLD. The study was approved by the Pediatric Regional Ethics Committee of Tuscany (No. 177/2017). All parents signed a written informed consent form for their participation.

### 2.2. Intervention

The mindful parenting intervention was based on the model developed by Bögels and Restifo [23] and was delivered in a group format. The program consisted of eight weekly sessions, each lasting approximately two hours. Groups were led by trained clinicians with expertise in mindfulness-based interventions and child psychopathology.

Each session included a combination of formal mindfulness practices (e.g., breathing meditation, body scan, mindful movement), experiential exercises focused on parenting situations, psychoeducational components, group discussions, and home practice assignments. Particular emphasis was placed on helping parents recognize automatic patterns of reaction in stressful parenting situations and cultivate greater awareness, emotional regulation, and compassion.

Importantly, the intervention was specifically adapted to address the relational and emotional regulation needs of children with neurodevelopmental disorders. Sessions included reflections on how ND-related characteristics (e.g., impulsivity, sensory sensitivities, rigidity) may influence parent–child interactions and how parents can adjust expectations and responses accordingly. Parents were encouraged to adopt a more flexible and compassionate stance toward both themselves and their children. A detailed description of the program structure and session content is provided in Muratori et al. [24]. Table 1 summarizes the in-session activities. Weekly homework assignments were subsequently introduced according to the topic addressed in each meeting and targeted three main dimensions: formal practice (e.g., body scan), informal practice (e.g., mindful coffee), and the cultivation of a beginner’s mind (e.g., approaching the child with curiosity rather than judgment).

### 2.3. Measures

Parenting practices were assessed using the Alabama Parenting Questionnaire (APQ) [25], a 42-item self-report measure. Items are rated on a 5-point Likert scale, with higher scores indicating greater endorsement of the respective parenting practices. In the current study, composite indices of positive parenting (parental involvement and positive parenting subscales) and negative parenting (inconsistent discipline, poor monitoring, and harsh discipline subscales) were used [26]. In the current sample, Cronbach’s alphas were 0.73 for positive parenting and 0.71 for negative parenting.

Dispositional mindfulness was assessed using the Five Facet Mindfulness Questionnaire (FFMQ) [27], a 39-item self-report instrument measuring five facets of mindfulness: observing, describing, acting with awareness, non-judging of inner experience, and non-reactivity to inner experience. Responses are rated on a 5-point Likert scale. Even though mindfulness facets are often considered traits, systematic reviews indicate that interventions can produce changes in personality-related characteristics [28]. Furthermore, the FFMQ has been widely used in mindfulness-based intervention research, including previous studies evaluating mindful parenting programs [29,30], supporting its use as an outcome measure in this context. In the current sample, Cronbach’s alphas were 0.79 for observing, 0.76 for describing, 0.82 for acting with awareness, 0.70 for non-judging of inner experience, and 0.72 for non-reactivity to inner experience.

### 2.4. Statistical Analysis

Analyses included only participants who completed the intervention, all of whom provided complete data at both baseline and post-intervention. Descriptive statistics were calculated for all variables at pre- and post-intervention. Paired-sample *t*-tests were conducted to examine changes in parenting practices and mindfulness facets from baseline to post-intervention. Effect sizes were estimated using Hedges’ g to correct for small sample bias. According to conventional benchmarks, Hedges’ g values of 0.20, 0.50, and 0.80 were interpreted as small, medium, and large effects, respectively.

## 3. Results

Table 2 shows means and standard deviations for all measures completed before and after the mindful parenting intervention and results of the paired sample *t*-tests. Negative parenting significantly decreased with a small effect size, whereas Acting with Awareness, Non-Judging, and Non-Reacting increased with small to medium effect sizes. No significant differences were found between pre- and post-measures of Positive Parenting, Observing, and Describing.

## 4. Discussion

The present pilot study examined the effects of a mindful parenting intervention on parenting practices and dispositional mindfulness among parents of children with neurodevelopmental disorders. Overall, the findings provide preliminary evidence that mindful parenting programs may be effective in this clinical population. Consistent with previous research [21,22], results suggest small-to-medium improvements in negative parenting and several dimensions of mindfulness (i.e., acting with awareness, non-judging, and non-reacting).

Among the findings, a reduction in negative parenting emerged as potentially meaningful from a clinical perspective. Parents reported lower levels of harsh, inconsistent, and maladaptive disciplinary practices following the intervention, consistently with previous studies showing improvements in parenting behaviors following mindful parenting interventions [15,16,31,32]. This result is particularly important given the robust evidence showing that children with ADHD, ASD, and related conditions often display behaviors—such as impulsivity, emotional dysregulation, rigidity, or oppositionality—that can elicit strong emotional reactions in caregivers [31]. Over time, these repeated stressors may foster automatic, reactive, and punitive parenting responses, which can inadvertently maintain or exacerbate children’s difficulties. From this perspective, the observed reduction in negative parenting may reflect parents’ enhanced capacity to interrupt these automatic cycles and respond in a more regulated and intentional manner. In line with this, the reduction in negative parenting does not necessarily imply a corresponding increase in positive parenting behaviors, which indeed was not detected in our study, but rather, it may reflect the mere inhibition of maladaptive or reactive behaviors.

The improvements observed in specific facets of mindfulness may offer insight into the potential mechanisms underlying these changes in parenting behavior. Acting with awareness increased significantly, suggesting that parents became more attentive and present during daily interactions with their children, rather than operating on “autopilot.” In the context of parenting a child with ND, acting with awareness may allow caregivers to better recognize early signs of stress or escalation—both in themselves and in their children—and to choose more adaptive responses. Similarly, increases in non-judging indicate a greater tendency to relate to internal experiences (e.g., frustration, guilt, feelings of inadequacy) with acceptance rather than self-criticism. This aspect may be especially relevant for parents of children with ND, who often report high levels of parental guilt, shame, and perceived failure. By cultivating a non-judgmental stance toward their own emotional experiences and their child’s behaviors, parents may reduce emotional reactivity and foster a more compassionate parenting approach.

Non-reactivity showed the largest effect size among the mindfulness facets. This exploratory finding aligns closely with theoretical models of mindful parenting, which emphasize parental self-regulation as a central mechanism of change. Non-reactivity refers to the ability to notice distressing thoughts and emotions without being overwhelmed or compelled to act on them immediately. For parents facing frequent behavioral challenges, this capacity may be crucial in preventing escalation during emotionally charged interactions. Rather than suppressing emotions or disengaging, parents may learn to tolerate discomfort and respond with greater flexibility and emotional balance. In turn, this may contribute to a more predictable and emotionally safe relational environment for the child.

No significant changes emerged in positive parenting practices or in the observing and describing facets of mindfulness. This pattern of results is consistent with previous intervention studies, which often report stronger effects on negative parenting and regulatory aspects of mindfulness than on positive parenting behaviors, e.g., [15,16]. One possible explanation is that positive parenting levels were already relatively high at baseline, leaving limited room for improvement. Alternatively, changes in positive parenting may require longer interventions, more intensive behavioral components, or extended time to consolidate and translate internal changes into observable parenting behaviors. Similarly, observing and describing may be less directly targeted by parenting-focused mindfulness programs compared to facets more closely related to emotional regulation and reactivity, and these facets have demonstrated reduced responsiveness across interventions, e.g., [33]. Moreover, the observing facet in particular has been shown to vary with meditation experience and often shows weaker associations with other mindfulness facets and outcomes in non-meditators [27,34]. This suggests that it may be less responsive to interventions for participants without prior meditation practice and may require more sustained or intensive practice to change. As for the describing facet, improvements may require explicit practice in noticing and labeling internal experiences, which is often only indirectly addressed in parenting-focused programs.

Overall, the findings offer preliminary indications that mindful parenting interventions may be particularly effective in enhancing parental self-regulation rather than directly modifying overt parenting behaviors in the short term. This distinction is important, as improvements in self-regulation may represent a foundational change that precedes and facilitates longer-term shifts in parenting style and parent–child relationship quality. In families of children with neurodevelopmental disorders, strengthening parents’ internal resources may be a necessary first step toward more sustainable and adaptive parenting practices. At the same time, considering that the intervention included both mindfulness practices and psychoeducational components, the observed changes in parenting may reflect the combined influence of these elements. Therefore, it is not possible to determine whether improvements were driven primarily by the mindfulness training, the educational content, or the interaction between the two.

A number of limitations should be kept into account when interpreting these findings. The primary limitation of this study is its single-group pre–post design, which restricts causal inferences; observed improvements could partly reflect nonspecific factors such as group support, expectancy effects, or regression to the mean. Second, the relatively small sample size reduced the statistical power and generalizability of the results. The limited sample size, along with the heterogeneity of child diagnoses, prevented us from examining how differences in diagnosis, symptom severity, or functional impairment may have influenced parental outcomes. Third, the exclusive reliance on self-report measures which may increase the risk of shared method variance and social desirability bias. While no additional measures were included in the present study, future research would benefit from incorporating observational or multi-informant assessments to provide a more robust evaluation of intervention effects. Moreover, the lack of follow-up assessments precludes conclusions regarding the durability of the observed changes over time. Also, approximately 20% of parents dropped out after the first two sessions. Due to the small sample size and limited data from dropouts, it was not possible to formally compare completers and drop-outs. Therefore, we cannot rule out the possibility of systematic differences between these groups, which may have influenced the findings. Finally, given the increased risk of type I error and the relatively small effect sizes, the practical and clinical significance of the findings should be interpreted with caution. Future research should build on these preliminary findings by employing randomized controlled designs, larger and more diverse samples, and multi-method assessment strategies, including parenting-specific measures of mindfulness and observational assessment of parent–child interactions. Longitudinal studies are also needed to examine whether changes in parental mindfulness and negative parenting translate into sustained improvements in child behavior and family functioning over time. Exploring potential moderators (e.g., child diagnosis, symptom severity, parental psychopathology) and mediators (e.g., reductions in parenting stress) may further clarify for whom and how mindful parenting interventions are most effective.

## 5. Conclusions

Mindful parenting interventions may represent a promising approach for supporting parents of children with neurodevelopmental disorders. By enhancing awareness, acceptance, and emotional regulation, such programs could help reduce maladaptive parenting practices and promote healthier parent–child relationships. The present preliminary findings contribute to the growing evidence base supporting the integration of mindful parenting interventions into clinical services for families of children with ND. Offering this kind of intervention in child psychiatry hospitals may be beneficial, as families are often overwhelmed and emotionally vulnerable. Helping parents understand their child’s needs and respond with greater compassion and self-regulation might contribute to improvements in the child’s recovery and the family’s well-being.

## Figures and Tables

**Table 1:** Description of the intervention’s meetings.

Session	Topic	Activities
1	Parental Stress	Parental stress and automatic parenting Body scan meditation
2	Beginner’s Mind	Parental expectations Sitting meditation
3	Reconnecting with Body	Attention to bodily sensations Awareness of pleasant moments Seated yoga
4	Responding to the Child Mindfully	Stressful situations and acceptance Standing yoga 3 min breathing practice as a coping strategy
5	Parenting Pattern	Parental schema Child ND and its impact on parent–child interactions and parenting Mindful parenting as a support for reducing parental reactivity Walking meditation
6	Conflicts and Parenting	Applying mindfulness to parental conflicts Oppositional behaviors and coercive cycles Mindful parenting to support emotion regulation during parent–child conflicts
7	Self-Compassion	Loving-kindness meditation Self-compassion practices Planning a family mindful day
8	Mindful Parenting	Body scan meditation Care plan for the child and the parent Consolidation of learning and future intentions

**Table 2:** Descriptive statistics at pre- and post-intervention and intervention outcomes (*N* = 50).

	T0		T1				
	***Mean***	***SD***	***Mean***	***SD***	***t***	***p***	Hedges’ g *
Positive Parenting	42.54	7.03	43.65	7.66	−1.74	0.090	0.15
Negative Parenting	10.49	3.88	9.32	4.10	2.82	0.008	0.29
FFMQ Observing	23.94	4.93	24.38	5.75	−1.10	0.143	0.11
FFMQ Describing	29.06	5.92	29.17	5.38	−0.17	0.868	0.01
FFMQ Acting with Awareness	27.54	6.63	29.03	4.89	−2.14	0.039	0.26
FFMQ Non Judging	26.46	6.62	28.63	5.86	−2.17	0.037	0.35
FFMQ Non Reacting	21.11	3.79	22.81	3.13	−2.68	0.011	0.52

* Hedges’ *g*: small effect = 0.20, medium effect = 0.50, large effect = 0.80.

## Data Availability

Data are available from the corresponding author upon reasonable request.
